# RETRACTION: LncRNA NKILA Promotes Epithelial-Mesenchymal Transition of Liver Cancer Cells by Targeting miR-485-5p

**DOI:** 10.1155/jo/9874094

**Published:** 2025-10-07

**Authors:** Journal of Oncology

RETRACTION: Y. Wang, C. Li, Y. Shi, and J. Kuai, “LncRNA NKILA Promotes Epithelial-Mesenchymal Transition of Liver Cancer Cells by Targeting miR-485-5p,” *Journal of Oncology* 2021 (2021): 1281031, https://doi.org/10.1155/2021/1281031.

The above article, published online on 03 September 2021 in Wiley Online Library (https://wileyonlinelibrary.com), has been retracted by agreement between the authors and John Wiley & Sons Ltd. The retraction has been agreed on request of the authors, due to concerns around the integrity of the results. After an investigation, it was found that the cell lines, “SMMC-7721” and “HL-7702,” have been contaminated [1]. As such, the results reported in the article are not considered to be reliable.
